# Supplementary material for: Offshore Wind Energy in India: Foundation Design and Challenges
Source: Indian Geotech J. 2026 Mar 18;56(9):4383–99. doi: 10.1007/s40098-026-01515-4 (PMC13602883; doi:10.1007/s40098-026-01515-4)
Supplement: Supplementary file 1 [file 40098_2026_1515_MOESM1_ESM.docx]

Reply to Reviewers Comments:

The author is grateful for the very kind comments provided by both the reviewers. All the comments were addressed and the manuscript was modified accordingly.

**Review #1**

| **Reviewer’s Comment** | **Author’s response** |
| --- | --- |
| 1. The abstract is generic. While most of the manuscript deals with centrifuge testing and monopiles, the abstract doesn't really focus on these aspects. The generic statements can be shifted to the Introduction and the essence of the centrifuge studies can be included. | The author agrees with the reviewer and modified the abstract to include the main impact of the centrifuge modelling for tests in clay and in liquefiable sands. |
| 2. The abstract talks about 3D numerical modelling, though the manuscript does not present 3D numerical analysis. | The reference to 3D modelling has been removed. |
| 3. Expand DNV in abstract and in text where it is appearing first. | DNV stands for Det Norske Veritas, and is now included in the manuscript. |
| 4. Fig. 1: Explicity cite the source in the caption, The copyright seems to be outdated. | Added the reference. |
| 5. Replace the phrase "loose, sand" with "loose sand" throughout the manuscript. | Done |
| 6. The abstract and the conclusions emphasize the OWTs in Gujarat, though the manuscript deals with Gujarat OWTs only to provide the information on their need, current proposals and challenges involved. Even the title of the paper includes India, though the presented results pertain to centrifuge tests carried out at Cambridge, not necessarily involving any date from India. Consider making appropriate changes to rightly reflect the contents in the title, abstract and conclusions. | The authors intention was that the developments in India both along the East Coast and West coast can be helped on the research that was carried out on clays and liquefiable sands at Cambridge. Lessons learnt from these studies can guide and direct research requirements in India. |
| 7. The resolution of Fig. 2 is poor. | Done. The figure has been redrawn to create a higher resolution figure. |
| 8. Fig. 3 is not cited in the text. | Done. Fig. 3 is now cited in the text. |
| 9. The symbols in axes titles and legends in most of the figures are not in italic font. | Done. |
| 10. In Figure 5, distingush the images as a &b and explain them in the caption. The centrifuge test setup is not completely explained. For example, PPTs need to be mentioned and expanded in text. | Done |
| 11. Line 183: "The increase .... is 1.67 times larger" needs correction. | Done |
| 12. Figure 10: Part 2, Part 3 and OWF 01RR need explanations. If some of these plots are not needed, remove them from the figure. | Explanation of OWF 01RR has been added. |
| 13. Figure 12: Expand PPT or explain in text | Done |
| 14. Line 362-363: Explain "Kobe motion". | Done. |
| 15. Fig. 18 & Fig. 19: Explain/remove sigma v - uhyd plot. | Done |
| 16. Shorten the conclusions section. | The author feels the conclusion section reads well. |
| 17. References do not follow the format of the journal. | References are corrected to be in the right format for IGJ |

**Review #2**

| 1. The overall technical approach and interpretation appears appropriate, but the manuscript would benefit from minor language polishing, particularly in sections describing methodology and results, to improve clarity and flow. | The author thanks the reviewer and the language has been polished. |
| --- | --- |
| 2. Figure 1 requires significant improvement. At present, it lacks adequate referencing and detailed map information. The authors are advised to redraw this figure with clearer geographic context, scale, North arrow, and proper citation, rather than relying on a generic or unreferenced image. | This Figure has been corrected and a correct reference has been added for the base picture. |
| 3. Figures 8 and 9 require revision in terms of labelling, resolution, and visual clarity. Axis labels, legends, and annotations are not sufficiently clear, which reduces the effectiveness of these figures in supporting the discussion. | These figures have been improved. |
| 4. The quality and consistency of figures across the manuscript should be reviewed carefully. While the figures are conceptually relevant, several appear to be prepared with limited attention to presentation standards expected for a journal publication. | The author agrees and improved the figures. |
| 5. Minor consistency checks are recommended for notation, symbols, and terminology related to offshore foundation behavior to ensure uniform usage throughout the manuscript. | Done. |
