# Supplementary material for: Offshore Wind Energy in India: Foundation Design and Challenges
Source: Indian Geotech J. 2026 Mar 18;56(9):4383–99. doi: 10.1007/s40098-026-01515-4 (PMC13602883; doi:10.1007/s40098-026-01515-4)
Supplement: Supplementary file 2 [file 40098_2026_1515_MOESM2_ESM.docx]

**Offshore Wind Energy in India – Foundation Design & Challenges**

**S. P. G. Madabhushi***

^*^Schofield Centre, Department of Engineering, University of Cambridge, High Cross, Madingley Road, Cambridge, CB3 0EF, UK, [mspg1@cam.ac.uk](mailto:mspg1@cam.ac.uk) (ORCID - [0000-0003-4031-8761](https://orcid.org/0000-0003-4031-8761))

^*^Corresponding author

**Abstract**

Offshore wind industry has made rapid advances in Europe particularly in the UK. Foundations for the Offshore Wind Turbines (OWT) form nearly 30% of the total cost and currently monopiles are the most popular form of these foundations. As the offshore wind industry moved to south-east asia including Japan, South Korea, Taiwan and China, the performance of OWT foundations under seismic loading has become important and there is much of current research in Cambridge directed towards this loading scenario. Offshore Wind is becoming popular in India with two sectors for wind farms already identified, one on the East Coast near Kanyakumari and the other offshore of Gujarat. In this paper, the design challenges for these two sectors will be considered. In the first part of the paper, the OWT foundations in the form of monopiles located in soft marine clays underlain by stiffer clay strata often present along the East Coast will be considered. The recent research using centrifuge modelling that is aimed at understanding the behaviour of monopiles under monotonic and cyclic loading will be highlighted. The results shows that realistic *p-y* curves can be generated directly from the centrifuge test data that capture the lateral monopile response in clays. In the second part of the paper the challenges of offshore wind development on the West Coast i.e. offshore of Gujarat will be emphasised. The recent research on earthquake loading and the seismic performance of monopile foundations in loose sandy soils overlying dense sandy strata will be considered. Recent design guidelines developed by Det Norske Veritas (DNV) that require the consideration of seismic load and operational wind/wave loading in tandem, and the recent research at Cambridge to test these load combinations will be highlighted. It will be shown based on centrifuge test data that the monopile response in liquefiable soils is significantly altered in the presence of operational wind/wave loading.

**Keywords:** centrifuge modelling, design, earthquakes, offshore wind, monopiles, numerical modelling

**Highlights**

- The paper highlights the development of offshore wind farms in India
- The design challenges faced for the offshore sectors identified along the East and West Coast of India are discussed
- Recent research results on monopile foundations in soft marine clays overlying stiffer clay strata are presented, considering both the pushover loads as well as operational cyclic loads.
- Additional challenges faced in the design of monopiles in sandy soil strata where liquefaction due to earthquake loading can occur, are discussed
- The modelling efforts that are able to consider important load combinations such as the earthquake loads in combination with the operational wind/wave loads are presented.

**1 Introduction**

The vast coastline of India offers excellent opportunities for development of offshore wind energy, that is now well recognised. Offshore windfarms yield more energy than onshore windfarms due to the consistent wind patterns offshore and unobstructed wind flow, which can offset the higher capex of these windfarms during the development phase. The success of offshore wind energy in Europe particularly in the UK, Germany, Denmark and other European countries in the last decade reflects the advantages of developing offshore wind farms. The offshore wind farm development in India is in early planning stages with two regions already identified as shown in Fig. 1 and marked in red and orange. The sites near southern coast of Tamil Nadu on the East Coast and the coastal areas near bay of Khambhat and Dwaraka in Gujarat were identified based on the high wind velocity (> 8m/s) for a significant period of the year as identified by surveys conducted by the National Institute of Ocean Technology, NIOT [1]. Further developments along the East Coast of India (Andhra Pradesh and Orissa) can be considered for development in the next phase where the wind velocity is moderate (> 6 m/s) for significant period of the year. These are marked in yellow in Fig. 1. Of course, further phases can be developed along the rest of the coastline in due course. NIOT have already mapped the wind velocity profiles along the entire coastline of peninsular India [1].

At many of the locations identified near Tamil Nadu and Gujarat (Phase I development) the water depths are < 50 m and therefore monopile foundations can be considered as suitable foundations for the Offshore Wind Turbines (OWTs) based on the experience in Northern Europe. The soil stratigraphy varies largely in these regions and also in regions for the future development offshore of Andhra Pradesh and Orissa (Phase II development). Often soft sediments i.e. marine clays are found at the sites underlain by stiffer clay or dense sand deposits. The monopile foundations for OWTs needs to penetrate through the soft deposit and terminate in the underlying in the denser deposit or rock-socket into the bedrock, which ever occurs first. An alternative type of soil stratigraphy may be loose, alluvial sand deposits that form the shallow layers that are underlain by dense sand deposits into which the monopile can terminate. In the sites offshore of Tamil Nadu, both these types of stratigraphy can be found. For the sites offshore of Gujarat, while these types of stratigraphy are found, there is an additional risk of seismic loading as witnessed in the Bhuj earthquake of 2001 [2]. In such cases the behaviour of the monopiles under the action of seismic loading needs to be understood.

***Fig. 1*** *Proposed Offshore Wind Energy sites (darker shade) and sites for future development (lighter shade) [Physical map of India, Courtesy: MapWire, 2026]*

In this paper the behaviour of monopiles in the two types of stratigraphy described above will be considered. In addition, the recent research on the behaviour of monopile foundations for OWTs under the action of seismic loading will be discussed, particularly when the loose sand layers are vulnerable from a liquefaction point of view.

**2. Design Principles of Monopile Foundations for OWTs**

Design of an offshore monopile foundations for OWTs is largely driven by the Serviceability Limit States (SLS) rather than Ultimate Limit States (ULS). The monopile foundations are subjected to vertical, horizontal and moment (V-H-M) loads. Typical gravity and environmental loads (wind and wave loading) are shown in Fig. 2 for a 5 MW wind turbine [3]. The vertical loads are relatively small but the horizontal loads due to wind and wave loading and the consequent moment loading at the seabed level are large.

**seabed**

***Fig.2*** Typical loading on an Offshore Wind Turbine [3]

These lateral loads and bending moments need to be resisted by the soil surrounding the monopile. The monopiles have relatively small length (*L*) to diameter (*D*) ratios with typical *L/D* ratios in the range of 2 ~ 4, compared to traditional piles that have *L/D* ratios of > 20. As a result, the monopiles are likely to undergo rigid body rotation rather than flexural bending, when subjected to lateral and moment loads at the seabed level.

**2.1 Monopile design drivers**

The monopiles are large steel, tubular piles with diameters in the range of 4 m to 8 m and more recently even larger pile diameters of 10 m and 12 m are being considered for supporting larger turbines. Fig. 3 shows the steel monopiles stocked on quay-side prior to deployment to the offshore sites.

***Fig. 3*** Example of monopiles on the dockside [Photo: Courtesy of Orsted]

The normal design of laterally loaded piles is based on p-y approach that links the lateral soil pressure (p) acting on the pile to the relative displacement between the pile and the soil (y), developed originally by Reece and Matlock [4]. These formed the basis for the American Petroleum Institute (API) method, however these were based on relatively small diameter piles. The API method was later adapted by Det Norske Veritas (DNV) standards, [5], [6], for large diameter monopiles as follows:

$p_{u}= \left\{ \left( 3 s_{u}+ \gamma^{'} X \right)+J \right.s_{u}X\}$ for $0<X<X_{R}$ …(1a)

$p_{u}= \left\{ 9s_{u}D \right\}$	for $X>X_{R}$ … (1b)

where $p_{u}$ is the ultimate soil pressure, $s_{u}$ is the undrained shear strength, *D* is the pile diameter, $\gamma^{'}$ is the submerged unit weight of soil, *X* is the soil depth below seabed, *J* is a dimensionless constant that ranges between 0.25 to 0.5. $X_{R}$ is transition depth where the value of $\left\{ \left( 3 s_{u}+ \gamma^{'} X \right)+J \right.s_{u}X\}$ > $\left\{ 9s_{u}D \right\}$ . In other words, the soil pressure $p_{u}$ is limited to $9s_{u}D$ which is the upper bound value on soil pressure for deep foundations. The above relations are useful for designing monopiles in clayey soils. More recently the PISA project in the UK developed more appropriate design equations for both sands and clays based on field testing of 2 m diameter piles, [7], [8] in Dunkirk sand and Cowden, UK respectively, and developed the PISA analytical methods for both marine sands and clays [9], [10].

***2.2 Natural frequency considerations***

Another aspect of monopile design is governed by the natural frequency of the monopile-foundation soil system. The driving frequencies of a 3 bladed wind turbine are identified as the frequency of a single blade passing in front of the tower (*1Ω*) causing turbulence due to air flow and the frequency of the three blades (*3Ω*) passing in front of the tower. Therefore, the natural frequency of the monopile-foundation soil system should avoid these frequencies. In Fig. 4 the nominal ranges of 1*Ω* and 3*Ω* frequencies along with the frequency ranges for wind and wave loading in the Ocean are shown. This leaves a fairly narrow range of frequencies between the 1*Ω* and 3*Ω* frequency bands available for the geotechnical engineers to target their monopile-foundation soil system’s natural frequency (marked in Fig. 4 as *f_n_*).

***Fig. 4*** Frequency ranges of the blade passing frequencies and the wind and wave loading.

Another aspect that needs to be considered is the natural frequency of the system is just above the 1*Ω* frequency. If the soil stiffness during the operational lifetime of the wind turbine were to degrade for any reason like excess pore pressure generation during an earthquake event, this may bring the natural frequency into the 1*Ω* frequency range causing the system to undergo resonant vibration [11]. The need to control the natural frequency of the OWT system, is one of the main reasons for the increase in the monopile diameters to 8 m and above, so that sufficient soil stiffness can be mobilised.

**3 Monopile foundations in clay**

Centrifuge modelling offers a quick and convenient way to model monopile foundations. The principles of centrifuge modelling and the scaling laws that relate model behaviour to the prototype are discussed by Madabhushi [12] and are not repeated here for brevity. In this section the testing of monopiles in clay soils will be discussed. A series of centrifuge tests were carried out at Cambridge as part of an EPSRC project to investigate the lateral capacity and cyclic behaviour of monopiles in clay layers [13], [14]. A 4 m diameter monopile was chosen as a prototype that were installed to a depth of 20 m, in normally consolidated clay profiles of different undrained shear strengths namely, 25 kPa, 50 kPa and 100 kPa. The centrifuge tests were carried out at 100g. A 2-D robotic actuator shown in Fig. 5 was used to apply the vertical and lateral loading. The monopiles were pre-installed at 1g for most of the depth, and on reaching the 100g sufficient time was allowed for reconsolidation of the clay. During this phase the monopile was allowed to settle with the clay during reconsolidation. This was made possible due to a sliding vertical connection between the actuator and the model monopile. Once the reconsolidation phase is complete, the monopiles were pressed-in by the robotic actuator for the last 1D (i.e. 4 m) of the monopile to reach the required installation depth. This enables the creation of the drive-in stresses during the installation process and mobilise the base capacity and skin friction of the monopile.

a) Cross-sectional view of the model
b) Assembled centrifuge model

***Fig. 5*** Centrifuge model of a monopile foundation in clay with the 2D robotic actuator [13].

The assembled centrifuge model is shown in Fig. 5 (a). As seen in Fig. 5 (b), the clay layer is instrumented with miniature Pore Pressure Transducers (PPTs) and piezo-electric accelerometers for shear wave velocity measurement. The model monopile was instrumented with MEMS accelerometers to measure inclination/rotation and with strain gauges at different elevations to measure bending moments as shown in Fig. 6.

***3.1 Push over tests***

In the initial phases of this research monopiles were simply subjected to lateral push-over loads. The horizontal load was measured using a load cell while the lateral displacements were measured using non-contact laser displacement transducers. In Fig. 6 the horizontal push over test results are presented for the three different clay profiles with undrained shear strengths of 25 kPa, 50 kPa and 100 kPa. The first feature of this data is that the stronger clays give higher lateral capacity as expected. However, it is important to notice that at a nominal horizontal displacement of 1 m, the horizontal capacity is ~1500 kN in a clay layer with $c_{u}$ = 25 kPa, which increases to only ~2500 kN in a clay layer with $c_{u}$ = 100 kPa. Although the strength has increased by four times, the increase in lateral capacity is only 1.67 times larger. This illustrates the non-linearity of the soil and emphasizes why centrifuge modelling is important for problems like this, where the non-linear behaviour of the soil needs to be captured accurately.

***Fig. 6*** Model monopile instrumented with strain gauges [13].

A further interesting feature is the limits imposed on the lateral displacement due the rotation limits of < 0.5^o^ imposed by DNV guidelines [5], [6]. This limits the lateral displacements in Fig. 7 to about 0.15 m (shown as shaded area in this figure). This area is expanded as an inset in Fig. 7. In the inset figure it can be seen that the initial stiffness during the lateral push-over is about 60 MN/m, which is almost the same for all the three clay strengths. As this initial stiffness is used in the estimation of the natural frequency calculation explained in previous section during the preliminary design process, monopiles in softer clay may be designed to have larger diameter than necessary. This will push the actual natural frequency of the system higher than calculated as during the normal operations the lateral displacements will be small for a larger diameter pile and therefore the stiffness will be higher at these displacement levels. Again, centrifuge test data can give valuable insights into the behaviour of the monopiles in clay and inform the design process and provide valuable economies of design. In Fig. 8 the soil heave observed in the $c_{u}$ = 50 kPa test is presented. The soil heave was captured numerically using ABAQUS software and presented previously by Haiderali et al [15] that matched very well with the observed soil heave in this figure.

***Fig. 7*** Push-over results for a 4 m diameter monopile in clay layers of different undrained strengths

***Fig. 8*** Soil heave in front of the monopile after the push-over test

During the push-over tests, the bending moments were recorded in the monopile at several levels. By using this data, it is possible to obtain experimentally the soil reaction variation with monopile displacement at different depths. The bending moment data can be double integrated to get the soil reactions (*p*) and double differentiated to get the relative pile-soil displacement (*y*). In Fig. 9 the experimentally obtained curves at depths of 4 m and 8 m are shown for the soil profile with $c_{u}$ = 100 kPa. For comparison, the design curves given by Eq. 1a and 1b) are plotted in the same graphs. At both these depths it can be seen that the initial stiffness obtained experimentally at small relative pile-soil displacements is higher than the design equations are predicting. Further the design equations are over-predicting the ultimate lateral capacity at larger relative pile-soil displacements. However, this is less of an issue as the design of monopiles is largely dictated by the initial stiffness rather than the ULS push-over capacity of the monopile.

a) at depth of 4 m

b) at depth of 8 m

***Fig. 9*** *p-y* curves for the 4 m diameter monopile in clay with $c_{u}$ = 100 kPa

These are example data shown here and similar procedures can be followed for more complex clay soil profiles that include over-consolidated clay layers at the surface or stiffer clay layers underlying the shallow, soft marine deposits near the seabed.

**3.2 Cyclic Loading**

In the second phase of this project the monopile was subjected to cyclic loading using the 2-D robotic actuator at 100g’s. The cyclic loading was applied under force-controlled conditions as the wind and wave loads will apply a lateral force on the monopile rather than imposing a displacement. A feedback loop was established to control the 2-D robotic actuator by measuring the horizontal load and applying incremental displacements to the actuator until the desired load is applied to the monopile. An example of the cyclic load application data is presented in Fig. 10.

***Fig. 10*** Target load-history achieved by the feed-back loop on application of incremental displacements

Wave packets of different amplitudes were applied to the monopiles. The details of each loading stage are shown in Table 1. The same wave packet configuration was used in all the centrifuge tests. The load amplitudes in each wave packets were increased from stage 1 to 6. Prototype loads are shown in kN within the brackets in Table 1. Both one-way loading and one and half-way loading (stage 4) were incorporated into the testing program, as previous research by Leblanc et al [16] has suggested that one and half-way loading may be more critical for monopiles than one way loading.

**Table 1** Wave packet configuration

| **Test Stage** | **Target Load (N)**  **(Prototype load in kN shown within brackets)** | **Number of Cycles** |
| --- | --- | --- |
| 1 | 10 (100 kN) | 100 |
| 2 | 50 (500 kN) | 328 |
| 3 | 100 (1000 kN) | 200 |
| 4 | -50 & 100 (-500 kN & 1000 kN) | 100 |
| 5 | 150 (1500 kN) | 110 |
| 6 | Monotonic Push | NA |

In Fig. 11 the experimental data is plotted directly at model scale for the centrifuge test OWF-05 that had a normally consolidated clay layer with $c_{u}$ = 50 kPa for different wave packets. In this data it can be seen that the amplitudes within each wave packet are being maintained constant by the 2-D actuator. The accumulation of the lateral displacements in each loading stage is also clearly seen. In Fig. 11, the backbone curve from the push over test is also shown. From this data it is clear that the horizontal displacements accrue within each wave packet although the applied horizontal load is smaller than that on the backbone curve. Further, the first few cycles in each wave packet seem to produce more displacement in the monopile, after which there is slower accumulation of the displacements in later cycles. This type of ratchetting behaviour can be very important in the accumulation of horizontal displacements and hence rotation of the monopile with cycles. During the design life of a monopile it is likely to see a number of storm loadings from waves and cyclones causing wind loading, and therefore it is important to understand the accumulation of the rotation. Centrifuge modelling offers a neat way of investigating these, prior to construction of a large number of OWT’s in the field.

***Fig. 11*** Accumulation of lateral displacement in each loading stage

The centrifuge test data on the lateral displacement from the loading stage 5 are plotted at model scale in Fig. 12 against the number of cycles. In this plot it can be seen that there is a bi-linear relationship in the semi-log space that can be established for the initial cycles (first 10 cycles) and the subsequent cycles (later 100 cycles). The correlation factors (R^2^ values) are very good for such a bi-linear fit. Using these relationships, the following set of equations can be written that link the approximate monopile rotation (θ) in radians to the number of cycles (*N*).

$\theta=0.0019 \ln(N)$	for $N\leq10$	… (2a)

$\theta=0.0044+0.0046 \ln(N-10)$	for $N>10$	… (2b)

Such equations can be useful in predicting the accumulated rotations after a certain number of extreme load cycles due to storm loading or cyclonic wind loading, at a given site.

***Fig. 12*** Bi-linear variation of the displacement with number of cycles

**4 Monopile foundations in sands subjected to earthquake loading**

Another possible soil stratification possible in offshore is loose sand layers that are underlain by dense sand deposits, as discussed in Sec. 1. Such deposits can be vulnerable to seismic loading with the shallow layers being susceptible to liquefaction. The recent DNV guidelines RP-0585 [17] recommend that the design of offshore monopiles should consider the anticipated seismic loading at the site, depending on the distance to the nearest fault, type of fault etc., in combination with the operational environmental loads due to wind and wave loading. This is justifiable given that the earthquake event can happen while the wind turbine is in operational mode. This load combination is likely to be the more onerous one in the design of monopiles. Also this poses some challenges in terms of centrifuge modelling that will be considered in this section.

Espanol-Espaniel et al [18] considered such a soil stratification for a large monopile foundation of 7.11 m diameter, as shown in Fig. 13. The Rotor-Nachelle Assembly (RNA) is modelled as a lumped mass on top of a tubular tower that is connected to the model monopile. The shallow, liquefiable layer of 7 m thickness was considered that was underlain by a dense sand layer of 15.4 m thickness that is located above the bedrock. The loose sand layer had a relative density (RD) of 41% while the deeper, dense sand layer had an RD of 82%. This sized monopiles are nominally used for a 10 MW turbine. In Fig. 13 the completely assembled centrifuge model that is loaded onto the centrifuge arm is also shown. The structural details of the monopile are presented in Table 2. The height of the tower above the monopile in the centrifuge model is shorter than in the field structure, due to limitations of the available height in the centrifuge. As a results the distance of the load eccentricity above the mudline is smaller, bringing the *e/D* ratio smaller. The natural period of the monopile-tower-RNA system however matches that of the field structure. Natarajan and Madabhushi [19] also investigated very similar soil stratification for the case of jacket structure supported on pin-piles of 2 m diameter under each of the jacket legs, with similar application and removal of lateral loading.

***Fig. 13*** Cross-sectional view of a large monopile centrifuge model in sand and a view of the model loaded onto the centrifuge

In order to consider the design load combination of seismic loading and operational wind/wave loading, a special setup was developed at Cambridge. The operational lateral load is estimated for the size of the wind turbine (10 MW in this case) and is applied at the top of the RNA lumped mass through a load cell and a steel wire. The steel wire passes through a pulley and a hanging mass that generates the required lateral load is applied at the other end of the wire. The hanging mass can be supported on a pneumatic piston in the extended position, which will remove the lateral load on the RNA mass. This is used during the swing up stage where the centrifugal acceleration is increased gradually to 70g. When the lateral load is to be applied prior to the earthquake shaking, the pneumatic piston is retracted in-flight, thereby applying the lateral load to the RNA mass. With this arrangement, the lateral load can be added or removed as desired between successive earthquakes.

**Table 2** Structural details of the monopile

| **Properties** | **Symbol** | **Prototype Structure** | **Actual Field Structure** |
| --- | --- | --- | --- |
| Diameter (m) | *D* | 7.11 | 7 – 12 |
| Embedded Length (m) | *L* | 17.5 | 25 – 40 |
| RNA height (m) | *e* | 46.28 | 150 – 200 |
| Aspect ratio | *L/D* | 2.46 | 3.5 – 5.5 |
| Load eccentricity ratio | *e/D* | 6.51 | 12.5 – 15 |
| Static moment at mudline (MNm) | *M_stat_* | 125 | 200 - 500 |
| Natural period (s) | *T_n_* | 4.5 (designed)  4.74 (with SSI effects) | 5.56 – 3.125 |

***4.1 Natural frequency of the tower-monopile system***

The natural frequency of the tower and the lumped mass of the system was designed to have a natural period of 4.74 s. However, this natural period is likely to increase due to the soil-structure interaction effects. To determine the actual natural period of the system, a small amplitude sine-sweep input motion of 0.025g was applied and the response of the RNA mass is measured experimentally. No lateral load was applied during this earthquake. The acceleration of the RNA mass is shown in Fig. 14. The corresponding response spectra are also plotted in Fig. 14. It can be seen that the natural period of the system is 4.74 s, which is longer than the designed fixed base period of the tower of 4.5 Hz. This period elongation is attributed to the soil-structure interaction (SSI) effects.

***Fig. 14*** Determination of the natural frequency of the tower-monopile system including the SSI effects

***4.2 RNA-Monopile response under weak and strong earthquakes***

The response of the RNA mass under weak and strong earthquake motions is considered next. First a weak, scaled-down Kobe motion was applied at the bedrock level. In Fig. 15 the input Kobe motion applied and the response of the RNA mass are presented. It can be seen that the RNA accelerations reach nearly 0.15g, a significant amplification compared to the input motion. Then a strong, sinusoidal input motion of 0.2g was applied at the bedrock level. The RNA accelerations reach nearly 0.5g under this motion. The corresponding response spectra for each of the input motions and the RNA mass accelerations are also shown in Fig. 15.

***Fig. 15*** Determination of the natural frequency of the tower-monopile system including the SSI effects

Clearly in both types of input motion significant amplifications are seen at the RNA level, highlighting the importance of considering the earthquake loading in this type of problems. These increased accelerations will be felt by the generation unit, gear box and other switch gear located within the RNA assembly. However, there is no resonance at 4.74 s period as seen earlier in Fig. 14 as neither of the input motions excite the system at this period.

***4.3 Rotation of the monopile***

One of the primary aims of this research is to determine if the earthquake input motions will result in the rotation of the monopile that exceeds the 0.5^o^ limit imposed by the DNV guidelines. In Fig. 16 the rotational response of the model monopile is presented for the scaled-down Kobe motion. The rotation of the monopile was determined by using the two LVDT recordings (see Fig. 13) and knowing the distance between them. Further, the applied dynamic moment was calculated using the load cell measurements and multiplying them with the height of the RNA above the mudline. This dynamic moment is also shown in Fig. 16. Prior to the application of the earthquake load, the static moment of 125 MNm applied to the system is also seen in Fig. 16. Under this earthquake loading the rotations are well below the ±0.5^o^ limits marked in Fig. 16 as dashed lines.

***Fig. 16*** Rotation of the monopile under scaled-down Kobe motion

In Fig. 17 the rotation of the monopile subjected to a much stronger 0.2g sinusoidal earthquake shaking considered earlier, is presented. Two separate shaking events are applied using this earthquake, one in the presence of a constant lateral load representing the environmental wind/wave loading and the other in the absence of the lateral load (calm air/calm sea condition). In both cases the applied dynamic moment above the mudline level are also shown in Fig. 17. The monopile undergoes a rotation of nearly 2^o^ in the presence of the lateral load, well above the DNV guideline limits. This is owing to the liquefaction of the shallow layer and reduction of soil stiffness in the dense layer under this stronger earthquake shaking. The excess pore pressures generated in these layers will be discussed later in this paper.

In the absence of the lateral load (i.e. the piston in extended position and supporting the hanging mass), the monopile suffered -1^o^ in the opposite direction, but still above the DNV guideline limits. This result suggests that the monopile is vulnerable to rotation with the onset of liquefaction, and the presence of lateral load exacerbates the rotation suffered by the monopile.

***Fig. 17*** Rotation of the monopile under strong sinusoidal motion

***4.4 Excess pore pressures in the shallow and deep sand layers***

The excess pore pressures that are generated in the shallow, loose sand layer and the deeper, dense sand layer are presented in Fig. 18 under the application of the 0.2g sinusoidal motion. In the free-field, away from the monopile foundation, it can be seen that the excess pore pressures reach near full liquefaction levels (i.e. *r*_u_ = 1). Even in the dense sand layer, at a depth of 17.15 m, the excess pore ratio reaches a value of *r*_u_ ~ 0.6, indicating significant reduction in the effective stress.

***Fig. 18*** Excess pore pressures away from the monopile

In Fig. 19, the excess pore pressures recorded at a depth of 2.52 m in the shallow sand layer, close to the monopile are presented for the case of earthquake loading in combination with the presence of lateral load. In this figure it can be seen that the excess pore pressure generation is very different on the windward side and the leeward side. On the windward side the excess pore pressures show a gradual buildup of positive excess pore pressures. On the leeward side, in contrast, the excess pore pressures show suction spikes. This data suggests that the application of the lateral load, changes the stress state of the soil on the windward side and leeward side of the monopile, that is manifested in the differences in the excess pore pressure generation. In Fig. 20, the excess pore pressures recorded on the windward and leeward sides of the monopile at a depth 3.56 m measured at a horizontal distance of 0.5*D* from the monopile, are plotted. In this figure the left hand plot shows the case where there is no lateral load applied to the RNA mass during shaking, while on the right hand side plot shows the case where lateral load was applied to the RNA mass in combination with the earthquake shaking. The input amplitudes of shaking in both cases was ~0.2g. Comparing the left and right hand side plots, the effect of the lateral load application is clearly seen. When there is no lateral load applied during shaking the excess pore pressures are positive and show gradual build up with the number of cycles of shaking. On the other hand, when the lateral load is applied during shaking, the excess pore pressure shows suction spikes superimposed with the excess pore pressure generation on the leeward side, while on the windward side it shows larger positive excess pore pressures with some suction spikes.

***Fig. 19*** Excess pore pressures close to the monopile

This difference in behaviour in terms of excess pore pressure generation on the windward and leeward sides under the application of the lateral load in combination of the earthquake shaking suggests the differences in the soil stress state in these regions of soil next to the monopile foundation. The application of the lateral load to the RNA mass to simulate the wind/wave load results in a difference in the horizontal effective stresses on the windward and leeward sides, which is being manifested as the difference in excess pore pressure generation discussed above.

***Fig. 20*** Excess pore pressures close to the monopile with and without lateral load application

From these centrifuge test results, it is possible to say that any fully coupled 3D finite element analyses of the monopile foundation problem, should also capture these differences in the windward and leeward side behaviour. That can be used in judging the efficacy of such numerical analyses. Similarly any analytical approaches that may be used in preliminary design of monopiles in liquefiable soils should also allow for different *p-y* springs on the windward and leeward sides. Development of such approaches are currently part of ongoing research at Cambridge.

**7 Conclusions**

In this paper some of the challenges faced in the design of monopile foundations for offshore wind turbines along the coastline of India are presented. Two specific soil stratigraphies are considered, firstly a normally consolidated soft clay marine deposits that overlay stiffer clays and secondly a loose, shallow sandy soil deposit overlying a denser, deep sand layer. Such soil stratigraphy can be identified in the currently identified sites near Tamilnadu and Gujarat states. The latter also has the possibility of seismic loading from future events like the Bhuj earthquake.

In the case of normally consolidated soft clay deposits, centrifuge testing was carried out on model monopiles that were instrumented with strain gauges. Using the data from these, the *p-y* springs were developed and compared to those proposed in the DNV guidelines. Some of the design considerations regarding the natural frequency of the monopile-soil system and the importance of determining the initial stiffness of the monopile-soil system are highlighted. Underestimation of the initial stiffness can lead to a larger monopile diameter not only increasing the cost of the foundation, but also results in a higher-than-expected natural frequency of the actual field structure.

In the case of sandy soil stratigraphy the main focus of this paper was on the earthquake induced liquefaction. The recent design guidelines from DNV indicate that the critical load combination of the operational wind/wave load should be considered along with the seismic loading. Novel centrifuge experiments were described wherein the lateral load could be applied or removed between successive earthquakes. The results from this research show that the application of lateral load can cause a difference in the soil behaviour on the windward and leeward sides of the monopile. Such an observation can have consequences in the design of the these monopiles and the tools used in the design. Fully coupled finite element analyses in the time domain should be able to predict the differences in the soil behaviour on the windward and leeward sides as seen in the experimental data. Similarly development of any simplified *p-y* springs should also account for these differences, perhaps using different spring on the windward and leeward side at every elevation of the monopile. Such approaches form directions for future research in this area.

**Declarations**

**Competing interests:** The authors have no competing interests to declare that are relevant to the content of this article.

**Funding:** The author declares that funding for some parts of the projects discussed in this paper came from EPSRC, UK (Grant No: EP/H013857/1) and Arup London.

**Data availability statement:** Data generated and analysed during this study are available from the corresponding author upon reasonable request.

**References**

[1] Alluri, S.K.R., Gujjula, D., Krishnaveni, B., Ganapathi, D., Phanikumar, S.V.S., Ramanamurthy, M.V. and Atmanand, M.A., (2018), Offshore wind feasibility study in India, in Stability Control and Reliable Performance of Wind Turbines, DOI: 10.5772/intechopen.74916.

[2] Madabhushi, S.P.G., D. Patel and Haigh, S.K., (2005), Geotechnical Aspects of the Bhuj Earthquake, Chapter 3, EEFIT Report, Institution of Structural Engineers, London, UK, ISBN 0901297 372.

[3] Byrne, B. W. & Houlsby, G. T. (2003). Foundations for Offshore Wind Turbine. *Philosophical Transactions: Mathematical, Physical and Engineering Sciences* **361**, No.1813, 2909-2930

[4] Reese, L. C. et. Matlock, H. (1960). Generalized Solutions for Laterally Loaded Piles*. Journal of the Soil Mechanics and Foundations Division, ASCE 86, No. 5, 1960, pp. 63 - 91.*

[5] DNV-GL. (2016). *Design of offshore wind turbine structures.* Offshore Standard DNV-OS-J101.

[6] DNV-GL. (2021). *Support structures for wind turbines*. Standard DNV-ST-0126.

[7] McAdam, R.A., Byrne, B.W., Houlsby, G.T., Beuckelaers, W.J.A.P., Burd, H.J., Gavin, K.G, Igoe, D.J.P., Jardine, R.J., Martin, C.M., Muir Wood, A., Potts, D.M., Skov Gretlund, J., Taborda, D.M.G. & Zdravković, L. (2020). Monotonic laterally loaded pile testing in a dense marine sand at Dunkirk. Géotechnique, <https://doi.org/10.1680/jgeot.18.PISA.004>.

[8] Byrne, B.W., McAdam, R.A., Burd, H.J., Beuckelaers, W.J.A.P., Gavin, K.G, Houlsby, G.T., Igoe, D.J.P., Jardine, R.J., Martin, C.M., Muir Wood, A., Potts, D.M., Skov Gretlund, J., Taborda, D.M.G. & Zdravković, L. (2020a). Monotonic laterally loaded pile testing in a stiff glacial clay till at Cowden. Géotechnique, <https://doi.org/10.1680/jgeot.18.PISA.003>.

[9] Byrne, B.W., Houlsby, G.T., Burd, H.J., Gavin, K.G, Igoe, D.J.P., Jardine, R.J., Martin, C.M., McAdam, R.A., Potts, D.M., Taborda, D.M.G. & Zdravković, L. (2020b). PISA design model for monopiles for offshore wind turbines: Application to a stiff glacial clay till. Géotechnique, <https://doi.org/10.1680/jgeot.18.P.255>.

[10] Burd, H.J., Taborda, D.M.G., Zdravković, L., Abadie, C.N., Byrne, B.W., Gavin, K.G, Houlsby, G.T., Igoe, D.J.P., Jardine, R.J., Martin, C.M., McAdam, R.A., Pedro, A.M.G. & Potts, D.M. (2020b). PISA design model for monopiles for offshore wind turbines: Application to a marine sand. Géotechnique, <https://doi.org/10.1680/jgeot.18.P.277>.

[11] Haigh, S.K. (2014). Foundations for Offshore Wind Turbines. Proceedings of the International Conference on Physical Modelling. (Eds. Gaudin. C & White, D.J.). ISBN 9781138001527.

[12] Madabhushi, S.P.G. (2014). *Centrifuge modelling for Civil Engineers.* CRC Press. ISBN 9780415668248.

[13] Lau, B.H., Lam, S.Y., Haigh, S.K. and Madabhushi, S.P.G., (2014), Centrifuge testing of monopile in clay under monotonic loads, Proc. 8th International Conf. on Physical Modelling in Geotechnics, UWA, Perth, Australia.

[14] Haiderali, A.E., Nakashima, M., Madabhushi, S.P.G., Lau, B.H. and Haigh, S.K., (2023), Numerical Modelling of the Monotonic Lateral Behaviour of XL and XXL Monopiles in Kaolin, *ASCE Journal of Geotechnical and Geo-environmental Engineering,* DOI:10.1061/JGGEFK/GTENG-11216.

[15] Haiderali, A.E., Cilingir, U. and Madabhushi, S.P.G. (2013). Lateral and axial capacity of monopiles for offshore wind turbines, Indian Geotechnical Journal, Vol. 43 (3), pp 181-194. DOI: 10.1007/s40098-013-0056-4.

[16] Leblanc, C., Houlsby, G.T. and Byrne, B.W. (2010). Response of stiff piles in sand to long-term cyclic lateral loading. Geotechnique, 60(2), pp. 79-90. <https://doi.org/10.1680/geot.7.00196>.

[17] DNV-RP-0585. (2021). *Seismic design of wind power plants recommended practice*.

[18] Espanol-Espaniel, C., Haigh, S.K., Madabhushi, S.P.G., Abadie, C.N., Go, J.E. and Morrison, P.R.J., (2024), Evolution of excess pore water pressures around monopiles subjected to moderate seismic loading, Journal of Soil Dynamics and Earthquake Engineering, 176, 108316.

[19] Natarajan K., Madabhushi GSP. (2022). Seismic Response of an Offshore Wind Turbine Jacket Structure with Pile Foundations, Soil Dynamics and Earthquake Engineering 162, DOI:[10.1016/j.soildyn.2022.107427](https://doi.org/10.1016/j.soildyn.2022.107427).
